# Supplementary material for: Can We Steer Nursing Home Residents Away from Loneliness? A Qualitative Study of Cycling without Age
Source: Geriatrics (Basel). 2024 Jun 25;9(4):88. doi: 10.3390/geriatrics9040088 (PMC11270177; doi:10.3390/geriatrics9040088)
Supplement: Supplementary file 1 [file geriatrics-09-00088-s001.zip › geriatrics-2987587-supplementary.pdf]

Supplementary Materials S1. Interview Guide:

Individual Semi-Structured Interviews with Nursing Home Residents

Individual interviews of nursing home residents, lasting 30-45 minutes.

Main Research Focus:
This study explores the experiences of nursing home residents who participate in Cycling Without Age (CWA) rides, with a particular focus on their perceptions of loneliness. It aims to understand the residents' lived experiences of the rides and how they influence feelings of loneliness.
The focus is on gaining insights into the informants' experiences, perspectives, reasoning, and ways of discussing the investigated phenomenon.

Follow-up, supplementary questions during the interview:

- Can you tell me a bit more about that?
- Can you describe how it went?
- When you say X, do you mean…?
- Can you elaborate on that?
- Can you give an example?
- REMEMBER, a pause for thinking is good!

Supplementary questions = Supp.

Theme	Research Question/Note	Operational Questions
Introduction before starting	Who we are and why we are here<br><br>Interview Structure<br><br>Anonymization and consent	My name is XXXX, and I am from the National Institute of Public Health.<br><br>With this interview, we aim to explore what it means for you to participate in Cycling Without Age. The interview is expected to last around 20-45 minutes.<br><br>I am interested in hearing your thoughts, feelings, and experiences, and there are no right or wrong answers. If you need a break during the interview, please let me know.

		I will record the conversation on this recorder. The recording is used to support my memory and will be included in the project work. The recording is treated confidentially, and the data will be deleted after the project concludes in 2027. In the project, your statements will be anonymized so that they cannot be traced back to you. Your rights are secured through the consent form you filled out before the interview.<br><br>Do you have any questions before we start?
Introduktion	Name, age, etc<br><br>Introductory Questions	I would like to get to know you a bit better first, so can you tell me a bit about yourself?<br>• Name<br>• Age<br>• Marital status/married/other.<br>• How long have you lived in the nursing home?<br>• How long have you been cycling with CWA?
Cycling Without Age	Subjective experience of participating in CUA:<br><br>*How can participation in Cycling Without Age help prevent and reduce loneliness among nursing home residents?*	• **Can you describe a typical day when you ride in the red cargo bike?**<br><br>• **How does it feel to be out cycling?**<br>o Supp: What do you feel before, during, and after the trip?<br>o Tell me about an experience you've had participating in Cycling Without Age?<br><br>• **Do you talk to others about participating in Cycling Without Age?**<br>o Supp.: Is it something you talk about at the nursing home or with others?<br>o What do you talk about when discussing the bike rides**?**<br><br>• **What does participate in Cycling Without Age mean to you?**<br><br>• **Have you ever not wanted to go cycling? If so, how did you feel after going?**<br>o Supp: Have you noticed any changes since you started cycling?

		○ How do you experience being part of the Cycling Without Age community?<br>○ How do you experience the conversations during the bike rides?<br><br>• **Do you feel you have made more friends or closer relationships by participating in Cycling Without Age?**<br><br>• **Do you sometimes ride with others?**<br>○ If yes: How do you experience the rides with others compared to riding alone?<br>○ If no: How is it to ride alone? Would you like to ride with someone else, and why?
Social Network from a Nursing Home Perspective	Subjective experience of living in a nursing home:<br><br>*How do nursing home residents perceive their social life and network?*	*I would like to start by hearing about how you experience living in a nursing home in terms of social life. (e.g., social circle, relationships, isolation, participation in activities)*<br><br>• **Can you describe what a good day is for you?**<br><br>• **Do you participate in the activities you would like to? -** Elaborate on why or why not?<br><br>• **How would you describe your social life?**<br>○ Supp: Who are your closest relationships? How often do you see them?<br><br>• **How has living in a nursing home affected you socially in terms of your social circle and how you interact with it?**<br>○ Supp: How do you experience that living in a nursing home has affected your relationships and friendships?

<table>
<tr><td>Loneliness</td><td>Subjective interpretation of loneliness:

How do participants in CUA experience loneliness?</td><td>Vignette: We know in advance that elderly people who move into nursing homes often feel lonelier compared to other times in their lives. Why do you think that is?

• What does loneliness mean to you?
    o Supp: Are there specific situations where you can feel lonely or not lonely in your daily life?

• If you have days where you feel lonely, does it affect whether you participate in activities, for example, bike rides?
    o Supp: And does it affect your social relationships? (Explain/elaborate/example)

• Do you have someone to talk to if there's something you find difficult or need to talk about?

• How has living in a nursing home affected your experience of loneliness?</td></tr>
<tr><td>Outro</td><td>Ensure a calm and positive end to the interview:</td><td>• How do you feel about me asking about loneliness?
• How has it been to participate in the interview? Was it okay?</td></tr>
</table>

Supplementary Materials S2. Observation Guide:

Observation Guide

**Theme**	**Where and When**	**Place, Date**	
		Descriptive Notes	Other: comments, intuition, analysis, and follow-up
Physical Space	• Furniture<br>• Objects<br>• Layout<br>• Surroundings<br>• Movements<br>• Smell<br>• Noise		
People Present	• Who is present<br>• What activities are they engaged in<br>• What are they saying		
Relationships Between People	Relationships between people present:<br><br>• Passenger : Care staff<br>• Passenger : Pilot<br>• Care staff : Pilot<br>• Other nursing home residents<br><br>Specific interactions between:<br><br>• People and space<br>• People and furniture<br>• People and objects<br>• Pauses, glances, touches, gestures		
